# Status of healthcare workers after comprehensive reform of urban public hospitals in Beijing, China: sustainable supply, psychological perception, and work outcomes

**DOI:** 10.1186/s12960-019-0421-1

**Published:** 2019-10-28

**Authors:** Jianwei Deng, Yangyang Sun, Run Lei, Yilun Guo, Jian Liu, Tianan Yang

**Affiliations:** 1 School of Management and Economics, Beijing Institute of Technology, 5 Zhongguancun South Street, Beijing, 100081 China; 2 Sustainable Development Research Institute for Economy and Society of Beijing, 5 Zhongguancun South Street, Beijing, 100081 China; 3 Beijing City Chaoyang District ShuangQiao Hospital, ShuangQiao East Road, Chaoyang District, Beijing, 100024 China; 4 Chair of Sport and Health Management, School of Management, Technical University of Munich, Uptown Munich Campus D, Georg-Brauchle-Ring 60/62, 80992 Munich, Germany

**Keywords:** Healthcare workers, Healthcare reform, Sustainable supply, Psychological perception, Quality of healthcare, Government data

## Abstract

**Background:**

Healthcare reform in China has attracted worldwide interest and reached a new juncture. In an attempt to improve healthcare quality and patient satisfaction, the government of Beijing introduced comprehensive reform of urban public hospitals in 2016 and implemented new policies on personnel, compensation, management, and diagnosis and treatment. As the agents of healthcare service, and a target of reform measures, healthcare workers were greatly affected by these reforms but have not been carefully studied.

**Methods:**

This study used mean value analysis, variance analysis, and qualitative content analysis to investigate the status of healthcare workers after comprehensive reform of urban public hospitals in Beijing.

**Results:**

We found a gradual but constant increase in the number of healthcare workers in poor health in Beijing public hospitals. After the reforms, this population reported high challenge stress, public service motivation, job satisfaction, job performance and quality of healthcare, moderate presenteeism, and low hindrance stress and turnover intention. The status of healthcare workers differed by subgroup and changed during the reform process.

**Conclusions:**

Our study provides data useful for policy recommendations regarding the implementation and extension of future reforms and offers important lessons for developing and developed countries that are reforming public hospitals to improve efficiency and reduce costs.

## Background

China is the most populous and one of the fastest-developing countries in the world, and healthcare reforms in China have justifiably attracted worldwide attention [1, 2]. The reform process has taken place in phases [3, 4]. During the first phase, from the late 1970s to 1992, the Chinese central government gave local governments responsibility for healthcare and initiated reforms creating a market-oriented medical system. Market-based reforms continued in the second phase, from 1992 to 2000, and resulted in reduced government economic input into the healthcare system, as well as decreased availability and increased cost of healthcare services and drugs [5]. During the third phase, from 2000 to 2005, faced with deteriorating access and rising expenditures, the Chinese government began to reflect on the drawbacks of market-oriented reform of the healthcare system and acknowledged that healthcare reform during the previous 10 years had failed [6]. To address the many persistent difficulties and challenges, China commenced a new round of healthcare reform in 2009 to increase healthcare affordability and strengthen primary care networks.

After successful implementation of the previous stage of reform, recent efforts have shifted to pilot reform efforts at “healthcare delivery organizations”, the most difficult task in the current healthcare reform [7]. Public hospitals in China account for almost 89% of hospital beds and 92% of hospital admissions [3] and thus have become the focus of current healthcare reforms. Beijing, the first pilot city for public-hospital reform, is central in any effort to improve healthcare. The pilot reform of public hospitals in Beijing started in 2010 and was expanded to all urban public hospitals in 2016, when the “implementation plan of comprehensive reform of Beijing municipal public hospitals” was issued [8]. The aim of this reform is to promote further reform of urban public hospitals and establish a healthcare service system consistent with a national capital. Experience gained during comprehensive reform of Beijing public hospitals will be utilized in nationwide reform efforts.

Since no further plans have been introduced in this comprehensive reform, it is urgent to evaluate existing policies, especially from the perspective of personnel management. Healthcare workers are the main agents of healthcare services and reform measures, and the primary communicators for patients [9].

Previous studies of healthcare reform focused on three aspects, as follows. (1) *Opportunities and challenges of healthcare reform* [1, 10]. For example, Papagianni maintains that the main challenges of healthcare reform are health disparities, socioeconomic inequality, rapid epidemiological transition, internal migration, and growth of aging populations. (2) *Achievement of medical reform and implementation of policy* [11, 12]. For example, He et al. conducted an empirical study to determine whether healthcare reform in China actually solved the issue of overly expensive healthcare services. (3) *Job satisfaction of healthcare workers before and after the reform* [9, 13]. For example, Ding et al. found that, after comprehensive healthcare reform, job satisfaction slightly improved among healthcare workers, but satisfaction was lower for the dimensions of pay, promotion, and benefits.

Therefore, previous studies of healthcare reform in China mainly used a macro perspective to focus on the challenges of reform or medical and economic outcomes, e.g., healthcare quality, patient satisfaction, and healthcare costs [3], and largely ignored the status of healthcare workers who have been negatively affected during the reform process—including their increasing work intensification and stress and deteriorating job quality and morale [4, 14, 15]—which are potential barriers to further reform. Although several studies have considered job satisfaction of healthcare workers before and after reform, comprehensive, in-depth research on the status of healthcare workers is limited.

To fill the gap between research and practice, this study investigated the effects of comprehensive reform of urban public hospitals in Beijing. Specifically, we used three measures to evaluate the status of healthcare workers qualitatively and quantitatively, as follows. (1) *Continuous supply of healthcare workers.* The shortage of healthcare workers and their poor health status are important obstacles in public-hospital development in China [16, 17]. To address this, we must increase the number of healthcare workers, while ensuring that they remain in their professions. Therefore, it is urgent to determine whether reform is helpful in ensuring the continuous supply and job stability of healthcare workers. (2) *Psychological perception of healthcare workers* was evaluated in relation to job stress, public service motivation (PSM), job satisfaction, and turnover intention. Healthcare workers are direct providers of healthcare service, and their psychological status may be an internal reason for a change in health care quality, so these variables are closely related to healthcare reform and to issues important to researchers [18, 19]. (3) *Work outcomes of healthcare workers*. *Presenteeism*, *job performance*, and *quality of healthcare* are the specific manifestations of work outcomes among healthcare workers, and work outcomes of healthcare workers largely determine the public’s direct feelings about healthcare. Moreover, these are important goals of healthcare reform [14, 20]. Finally, we suggest measures to promote reform of urban public hospitals in Beijing, from the perspective of personnel management.

Our study would have some potential theoretical and practical contributions. First, this study enriches research on the performance of healthcare reform from the perspective of personnel management rather than from economic and customer orientation perspectives. Second, this study provides empirical evidence likely to be valuable for future comprehensive reform of urban public hospitals in Beijing and nationwide. Third, pilot public-hospital reform in China has been comprehensive, but the challenges and experiences of the reform process are not unique and may prove more informative—as compared with OECD models developed in very different contexts—for developing countries [21] and developed countries in which public hospitals represent a large share of overall healthcare spending and are thus targets for efficiency and cost reforms [3].

## Methods

### Sampling and data collection

To evaluate the effects of comprehensive reform of urban public hospitals in Beijing on healthcare workers, we first obtained government statistical data (e.g., number of healthcare workers, hospital beds, and outpatient and inpatient numbers) from the website of the Beijing Municipal Health Commission Information Center (http://www.phic.org.cn/tjsj/wstjjb/).

Then, after obtaining ethical approval and informed consent, we conducted a cross-sectional questionnaire survey of 572 healthcare workers in six representative Beijing public hospitals (primary, secondary, and tertiary) from 10 October in 2017 to 30 January in 2018 (Table 1). According to the data, the ratio of primary, secondary, and tertiary hospitals in Beijing is approximately 4:1:1 (data from the Beijing Municipal Health Commission Information Center). Thus, we selected six representative hospitals, including four first-level hospitals, one secondary hospital, and one tertiary hospital. Using random selection by worker identification number, we selected 5 to 10% of healthcare workers from each chosen hospital. Ultimately, 572 questionnaires were included from 710 voluntary participants (response rate, 80.6%). In China, “healthcare workers” refer to all levels of health technicians who have been approved and recognized by the health administration department and have obtained corresponding qualifications and practicing certificates. The category includes clinicians (resident physicians, attending physicians, deputy chief physicians, and chief physicians), pharmacists, nursing staff (licensed nurses), and other technical personnel. In addition, because public hospitals in China have not implemented a professional management system, some senior clinicians and nursing staff also have administrative titles and responsibilities. During the study, we reminded participants with administrative titles to identify themselves as managers.

**Table 1:** Demographic characteristics of questionnaire respondents (*N* = 572)

Characteristics	Percentages
Sex	(1) Male (35.0%); (2) Female (59.6%)
Age (years)	(1) ~ 25 (7.5%); (2) 25~30 (27.3%); (3) 31~35 (26.0%); (4) 36~40 (15.0%); (5) 41~45 (8.4%); (6) 46~50 (7.0%); (7) 51~55 (3.7%); (8) 56~60 (3.1%)
Post	(1) Clinician (39.2%); (2) Nurse (34.6%); (3) Manager (2.3%); (4) Medical technician (11.9%); (5) Pharmacist (8.3%)
Education	(1) Under degree (5.9%); (2) Junior college (20.5%); (3) Undergraduate (40.0%); (4) Master’s degree (19.1%); (5) Doctorate (12.6%)
Title	(1) Primary (43.9%); (2) Middle (40.2%); (3) Senior (10.8%)
Seniority (years)	(1) ~ 3 (17.5%); (2) 3~5 (22.4%); (3) 6~10 (27.6%); (4) 11~20 (19.1%); (5) 20~ (11.2%)
Department	(1) Physician (27.1%); (2) Surgeon (14.2%); (3) Obstetrics/Gynecology (10.5%); (4) Pediatrics (1.9%); (5) Chinese Medicine (6.3%); (6) Emergency Department/Intensive care unit (3.1%); (7) Oncology (3.3%); (8) Other clinical departments (8.4%); (9) Medical Technology (5.8%); (10) Administration and Logistics (4.5%); (11) Other (12.4%)

Demographic information was missing for a small number of participants (1.9–5.4%)

The questionnaire included items on health, psychological perception, and work outcomes of healthcare workers and changes in related variables after the reform program. To guarantee data integrity and objectivity, we used work identification numbers to randomly select 5 to 10% of healthcare workers in each target hospital.

Finally, we analyzed data from in-depth interviews. A coordinator working at each hospital was responsible for contacting five to eight selected participants (a total of 36 participants). A wide range of characteristics was considered when selecting interviewees, including post, age, job title, sex, and seniority. During the interview, participants were asked to describe the status of and changes in their health, psychological perceptions, and work outcomes. Additionally, the demographic characteristics of respondents were analyzed.

### Measurement instruments

Health was assessed by using the 8-item Short-form Health Survey Scale (SF-8 general health), which analyzes the same eight health domains as the Short-form Health Survey 36 (SF-36) [22]. The items ask respondents to assess their health by using a 6-point Likert scale (1 = excellent, 6 = very poor) and 5-point Likert scale (1 = not at all, 5 = could not do daily activities). We reversed the scoring of these items, so that higher values reflect better health status. The scale had high reliability in our research (*α* = 0.89).

Job stress was measured with an 11-item Challenge and Hindrance-related Self-Reported Stress (C-HSS) scale [23], which had high reliability in our research (*α* = 0.85–0.81). The items ask respondents to evaluate their challenge stress and hindrance stress on a scale from 1 (no stress) to 5 (greatest stress). Higher values indicate greater turnover intention.

PSM was measured with the Short-form PSM scale [24], which has been used by numerous researchers in public administration and had high reliability in our research (*α* = 0.91). A 5-point Likert scale (1 = strongly disagree, 5 = strongly agree) was used to evaluate PSM of respondents. Higher values indicate greater PSM.

Job satisfaction was measured with a six-item scale [25], which had acceptable reliability in our research (*α* = 0.86). In this assessment, the items were used to evaluate job satisfaction of healthcare workers. A 5-point Likert scale (1 = strongly disagree; 5 = strongly agree) was used to express job satisfaction. We reversed the scoring of three items (JB2, JB4, JB6), so that higher values reflect greater job satisfaction.

Turnover intention was measured with a three-item measure developed by Singh et al. [26], which was confirmed to have high reliability in our research (*α* = 0.82). The items ask respondents to rate their turnover intention on a scale from 1 (strongly disagree) to 5 (strongly agree). Higher values indicate greater turnover intention.

Presenteeism was assessed with the four-item Perceived Ability to Work Scale (PAWS), which was reported to be a valid instrument for measuring perceived productivity loss [14]. The items ask respondents to assess their work ability by using a scale from 0 to 10 (0 = cannot currently work at all; 10 = work ability is currently at its lifetime best). We reversed the scoring of these items, so that higher values reflect greater presenteeism. The scale had high reliability in our research (*α* = 0.87).

Job performance was assessed with a four-item scale [27], which had high reliability in our research (*α* = 0.85). The items ask respondents to evaluate personal job performance and job performance in comparison with coworkers on a 5-point Likert scale (1 = strongly disagree; 5 = strongly agree). Higher values indicate greater job performance.

The quality of healthcare was assessed with the 13-item Chirurgisches Qualitätssiegel Scale [28]. The items ask respondents to rate their perceived healthcare quality on a scale from 1 (bad) to 5 (very good). Higher values indicate greater quality of healthcare. The scale had high reliability in our research (*α* = 0.94).

### Quantitative analysis

SPSS 20.0 was used for statistical analyses comprising descriptive analysis and analysis of variance. To determine if the effect of comprehensive reform on healthcare workers differed by group characteristics of healthcare workers, we categorized respondents by demographic characteristics. To ensure that subgroups were of equal size, hospital level was categorized as primary, secondary, and tertiary. Post was classified as physician, nurse, and other (e.g., medical technician and pharmacist). Age was categorized as old (41 years or older), middle (31–40 years), and young (30 years or younger). Job title was classified as early career (trainee or entry-level worker) and mid/late career (mid-level or senior worker). Sex was categorized as male and female. Seniority was classified as shorter than 5 years (employed for less than 5 years) and longer than 5 years (employed for longer than 5 years).

### Qualitative analysis

After obtaining participant consent, interviews were audio recorded and transcribed verbatim [1]. Three student assistants verified the accuracy of the transcripts. After that, one of the authors experienced in qualitative research classified and coded the data. Finally, we analyzed the coded data to verify the results of quantitative analysis.

## Results

### Sustainable supply of healthcare workers in Beijing public hospitals

From 2011 to 2017, the number of healthcare workers steadily increased in Beijing public hospitals (Table 2). After the pilot reform, the number of healthcare workers in Beijing public hospitals increased from 140 304 in 2011 to 198 290 in 2015, an average annual growth rate of 9.1%. In 2016, the Beijing government extended the pilot reform to all public hospitals in the city and augmented the reform objectives and tasks. The number of healthcare workers in Beijing public hospitals continued to increase, but the annual growth rate declined to 1.4%. This effect is reflected in the numbers of health technicians, licensed (assistant) physicians, and registered nurses (Table 2, columns 3–5).

**Table 2:** Changes in the numbers of healthcare workers in Beijing public hospitals, 2011–2107

	Healthcare worker	Health technician	Licensed (assistant) physician	Registered nurse
2011	140 304	111 900	38 873	51 425
2012	173 246	143 119	48 735	71 054
2013	183 889	148 653	50 706	74 049
2014	189 708	157 214	53 024	77 342
2015	198 290	165 610	57 304	83 148
2016	199 422	167 932	59 154	83 741
2017	204 048	171 803	60 395	85 785

Source: Information center of Beijing health and family planning commission, http://www.phic.org.cn/tjsj/wstjjb/

The means (SD) for the eight health items were low, and the range was moderate—from 3.24 (1.21) to 3.88 (0.93) (Table 3)—perhaps because healthcare workers in Beijing public hospitals were in poor health. During the interview, the interviewees repeatedly expressed concerns regarding their health status.

Although we attach great importance to our health and have professional knowledge of health, we’re continually overworked, with high mental stress and insufficient rest time. As a result, many healthcare workers in our hospital suffer from physical or mental diseases every year, and this problem seems to have worsened in the past few years. (P22, female manager, age 45 years).

I am a nurse and frequently work at night. This leads to poor sleep quality and insomnia at night, which affects my physical resistance. (P7, female nurse, age 26 years).

I often need to receive 80-90 outpatients; this busy working condition makes my diet irregular. Therefore, I have a serious stomach illness and lumbar disc herniation. Moreover, these two conditions are common among doctors. (P27, male clinician, age 34 years).

**Table 3:** Means (SD) for questions on health status (H)

Variable	Item	Mean	SD
Health (1–8)	H1. Overall, how would you rate your health during the past 4 weeks?	3.55	0.93
	H2. During the past 4 weeks, how much did physical health problems limit your physical activities (such as walking or climbing stairs)?	3.85	0.96
	H3. During the past 4 weeks, how much difficulty did you have doing your daily work, both at home and away from home, because of your physical health?	3.88	0.93
	H4. How much bodily pain have you had during the past 4 weeks?	3.24	1.21
	H5. During the past 4 weeks, how much energy did you have?	3.47	1.01
	H6. During the past 4 weeks, how much did your physical health or emotional problems limit your usual social activities with family or friends?	3.74	0.96
	H7. During the past 4 weeks, how much have you been bothered by emotional problems (such as feeling anxious, depressed or irritable)?	3.64	0.93
	H8. During the past 4 weeks, how much did personal or emotional problems keep you from doing your usual work, school or other daily activities?	3.76	0.94

### Psychological perceptions of healthcare workers in Beijing public hospitals

The means for challenge stress items were higher than those for hindrance stress items in Beijing public hospitals after the comprehensive reform (Table 4). Specifically, the means (SD) for the six challenge stress items were high, and the range was limited: from 3.37 (0.85) to 3.46 (0.89). In contrast, the means for the five hindrance stress items were lower, and the range was wider—2.43 (1.12) to 3.37 (0.85). These results were consistent with our expectations, as healthcare workers are typically under considerable job stress [29]. During the interview, many interviewees mentioned that they are under considerable job stress.

I’m under incredible job stress right now—from many sources—long work hours, heavy workload, low income, demanding research work, an imbalance between work and home, limited opportunities for advancement, and even misunderstandings with patients. Fortunately, my relationships with colleagues and managers are relatively simple (P13, female clinician, age 29 years).

I spend a lot of time on patients and scientific research every day, and I also need to take care of my family. Balancing work and family creates a lot of pressure. At the same time, I need to keep my mind focused for a long time and not make any mistakes. Otherwise, it will endanger the health and life of patients, which also makes me feel stressed. (P5, female clinician, age 39 years).

Frequent medical violence makes me feel lack of job security and professional responsibility, and is a psychological burden, which makes me unhappy at work. (P11, female pharmacist, age 24 years).

**Table 4:** Means (SD) for challenge stress (CS) and hindrance stress (HS) items

Variable	Item	Mean	SD
Challenge stress (1–6)	CS1.The number of projects and or assignments I have.	3.37	0.85
	CS2. The amount of time I spend at work.	3.45	0.86
	CS3. The volume of work that must be accomplished in the allotted time.	3.41	0.86
	CS4. Time pressures I experience.	3.39	0.85
	CS5. The amount of responsibility I have.	3.46	0.89
	CS6. The scope of responsibility my position entails.	3.39	0.87
Hindrance stress (1–5)	HS1. The degree to which politics rather than performance affects organizational decisions.	2.68	1.14
	HS2. The inability to clearly understand what is expected of me on the job.	2.43	1.12
	HS3. The amount of red tape I need to go through to get my job done.	3.09	1.01
	HS4. The lack of job security I have.	2.91	1.12
	HS5. The degree to which my career seems stalled.	2.94	1.10

Healthcare workers in Beijing public hospitals had very high PSM after the comprehensive reform (Table 5). The means (SD) for PSM items ranged from 3.67 (1.05) to 4.07 (0.80). High PSM values indicate that healthcare workers in Beijing public hospitals pay considerable attention to public values and public interest, not just personal interests. We reached similar conclusions during our interviews.

I’d rather work in public hospitals than in private hospitals. Public hospitals provide healthcare services to more patients, especially those with low incomes, so my job is valuable, which makes me happy and proud. And those feelings have increased with the development of public-hospital reform (P30, female nurse, age 42 years).

Public healthcare is a basic public service, which relates to people’s health. Therefore, I think my work is valuable and meaningful (P18, male medical technician, age 27 years).

It is said that, “love whatever job one takes up”. Since I choose to be a nurse, I will put the interests of patients in a very important position and try my best to provide good services for patients, so that patients are satisfied (P33, female nurse, age 40 years).

**Table 5:** Means (SD) for public service motivation (PSM) items

Variable	Item	Mean	SD
Public service motivation (1–5)	PSM1. Meaningful public service is very important to me.	4.07	0.80
	PSM2. I am often reminded by daily events about how dependent we are on one another.	3.96	0.88
	PSM3. Making a difference in society means more to me than personal achievements.	3.89	0.93
	PSM4. I am prepared to make sacrifices for the good of society.	3.67	1.05
	PSM5. I am not afraid to go to bat for the rights of others, even if it means I will be ridiculed.	3.68	1.04

The means (SD) for the six job satisfaction items were relatively high, and the range was wide, from 3.48 (1.20) to 3.97 (0.86) (Table 6). High job satisfaction was reflected in the questionnaire data and during interviews. During the interview, many interviewees mentioned that they are satisfied with their present job.

Although there are a lot of problems, I’m still satisfied with my job. I get to use my professional knowledge and the work has value (P17, male clinician, age 38 years).

My job satisfaction is relatively high overall. On a scale of 10, I would say it’s 8 to 8.5 points. My parents and relatives also agree with my job (P3, male manager, 34 years).

I am quite satisfied with my present job. My hospital is a well-known hospital, where I can learn a lot of new things and get clinical experience. In addition, I’m satisfied with my current salary (P9, female clinician, 28 years).

**Table 6:** Means (SD) for job satisfaction (JS) items

Variable	Item	Mean	SD
Job satisfaction (1–5)	JS1. My job is very pleasant.	3.68	0.90
	JS2. My job is very worthwhile.	3.97	0.86
	JS3. My job is better than most.	3.68	0.97
	JS4. I sometimes feel my job is a waste of time.	3.48	1.20
	JS5. I am very content with my job.	3.72	0.91
	JS6. This job is worse than most.	3.58	1.15

The means (SD) for the three turnover intention items were very low, and the range was small, from 2.34 (0.89) to 2.42 (0.93) (Table 7). The findings from interviews were similar: more than 85% of respondents said they had no short-term desire to change jobs. During the interview, many interviewees mentioned they intend to stay at their job.

Working in a Class A tertiary hospital is my career goal. There are good technical platforms, experimental equipment and well-known medical experts, which helps me improve my professional skills and develop my career (P4, male clinician, age 32 years).

Although there are many shortcomings and unsatisfactory aspects of the job, I feel that I can overcome these challenges and do not intend to leave my present job. At the same time, changing jobs in Beijing costs a lot, for example, time cost, material cost, etc. (P35, female manager, age 33 years).

I have no intention of leaving my job for the time being. I hope to improve my skills and expand my interpersonal network first. In the future, if there is a chance, I hope I can start my own business (P25, male medical technician, age 23 years).

**Table 7:** Means (SD) for turnover intention (TI) items

Variable	Item	Mean	SD
Turnover intention (1–3)	TI1. It is likely that I will actively look for a new job next year.	2.42	0.93
	TI2. I often think about quitting.	2.41	0.95
	TI3. I will probably look for a new job next year.	2.34	0.89

### Work outcomes of healthcare workers in Beijing public hospitals

The means (SD) for the four presenteeism items were moderate and similar (Table 8) and ranged from 3.92 (2.33) to 4.12 (2.38), which indicates some loss of productivity among healthcare workers in Beijing public hospitals. During the interview, many interviewees mentioned diminished productivity.

To be honest, I really can’t guarantee that I’ll be at my best when I’m at work. Sometimes my productivity and ability are down. Although it isn’t frequent, it does happen (P2, male clinician, age 42 years).

Physical illness and psychological stress can make it difficult for me to fully engage in my work. I need to constantly adjust. Of course, this is not common. It happens about 1 - 2 times a month (P1, male clinician, age 48 years).

I just got a promotion this year. I feel that my working ability and experience are still insufficient. I need to learn more and accumulate more in my future work (P36, male clinician, age 36 years).

**Table 8:** Means (SD) for presenteeism (P) items

Variable	Item	Mean	SD
Presenteeism (1–4)	P1. How many points would you give your current ability to work?	3.98	2.50
	P2. Thinking about the physical demands of your job, how do you rate your current ability to meet those demands?	4.12	2.38
	P3. Thinking about the mental demands of your job, how do you rate your current ability to meet those demands?	4.08	2.47
	P4. Thinking about the interpersonal demands of your job, how do you rate your current ability to meet those demands?	3.92	2.33

The means (SD) for the four job performance items were high, and the range was limited: from 4.01 (0.74) to 4.06 (0.75) (Table 9). We reached similar conclusions during the interviews.

During outpatient clinic hours, I need to see about 100 to 120 patients a day in this tertiary hospital, which increases my job performance. Also, performance evaluation is an important part of this reform, so I have to really pay attention to job performance (P34, female clinician, age 48 years).

I think my job performance is still relatively high, because I am familiar with my work and my colleagues have also given me a lot of help (P6, female nurse, 23 years).

The hospital has very strict performance appraisal standards; therefore, our work performance is high. Otherwise, our income will be affected and we will not be able to complete our daily tasks (P14, male clinician, 29 years).

**Table 9:** Mean (SD) for job performance (JP) items

Variable	Item	Mean	SD
Job performance (1–4)	JP1. Quality of your performance.	4.03	0.71
	JP2. Your productivity on the job.	4.06	0.75
	JP3. How do you evaluate the performance of your peers at their jobs compared with yourself doing the same kind of work?	4.01	0.74
	JP4. How do you evaluate the performance of yourself at your job compared with your peers doing the same kind of work?	4.05	0.76

The means for the 13 quality of healthcare items were relatively high, and the range was wide—from 3.53 (1.10) to 4.03 (0.81) (Table 10). These findings indicate that the quality of healthcare provided by healthcare workers in Beijing public hospitals was high. During the interview, many interviewees mentioned that their perceived quality of healthcare is relatively high.

The medical staff at our hospital have good professional qualifications and rich clinical experience and have formed an integrated diagnosis and treatment system, as well as an expert consultation system. Also, the hospital has constantly improved the healthcare quality control processes and systems during the comprehensive reform, which ensures healthcare quality (P21, male manager, age 43 years).

The quality of healthcare service in our hospital is very good, and the patients are satisfied. In the future, we will further improve the quality of healthcare service (P31, male clinician, 37 years).

I’m confident of the service quality I provide. For each patient, I ask about their condition and history in detail, examine them carefully, and make good medical records (P24, female clinician, 43 years).

**Table 10:** Means (SD) for quality of healthcare (QH) items

Variable	Item	Mean	SD
Quality of healthcare (1–13)	QH1. Perform surgeries.	3.53	1.10
	QH2. Assess diagnostic information.	3.88	0.83
	QH3. Make correct diagnoses.	3.92	0.80
	QH4. Select appropriate treatments.	3.91	0.87
	QH5. Maintain medical records.	4.00	0.82
	QH6. Inform patients about rationale for treatment.	4.00	0.85
	QH7. Consider psychosocial aspects of illness.	3.96	0.84
	QH8. Manage health care resources efficiently.	3.99	0.82
	QH9. Evaluate medical literature to optimize clinical decision-making.	3.90	0.89
	QH10. Participate in implementation of quality improvement programs.	3.85	0.84
	QH11. Show empathy for patients and their relatives.	4.03	0.81
	QH12. Involve patients in decision-making.	3.88	0.82
	QH13. Consider advance health care directives.	4.01	0.79

### Differences among healthcare worker groups in Beijing public hospitals

Tables 11 and 12 show that values regarding health, challenge stress, hindrance stress, PSM, job satisfaction, turnover intention, presenteeism, job performance, and quality of healthcare differ in relation to hospital level and demographic characteristics of healthcare workers. For example, health, challenge stress, hindrance stress, job satisfaction, and turnover intention significantly differed among employees of primary, secondary, and tertiary hospitals. Presenteeism differed in relation to post, hospital level, age, job title, and sex but not in relation to seniority. PSM, job performance, and quality of healthcare differed in relation to post, hospital level, age, job title, and seniority but not in relation to sex.

**Table 11:** Differences in all variables’ items among healthcare worker subgroups (part 1)

	Post			*P*	Hospital level			*P*
	Clinicians (*n* = 224)	Nurses (*n* = 198)	Others (*n* = 117)		Primary (*n* = 120)	Secondary (*n* = 256)	Tertiary (*n* = 196)	
H	3.63 (0.81)	3.61 (0.75)	3.82 (0.84)	–	3.81 (0.81)	3.72 (0.83)	3.43 (0.71)	***
CS	3.47 (0.64)	3.41 (0.85)	3.32 (0.73)	–	3.40 (0.58)	3.30 (0.83)	3.56 (0.70)	**
HS	3.78 (0.79)	2.82 (0.99)	2.82 (0.83)	–	2.99 (0.61)	2.74 (1.04)	2.79 (0.77)	*
PSM	3.82 (0.71)	3.78 (0.81)	4.07 (0.85)	*	3.60 (0.62)	3.87 (0.90)	4.00 (0.76)	***
JS	3.74 (0.67)	3.64 (0.70)	3.69 (0.61)	–	3.53 (0.61)	3.72 (0.70)	3.74 (0.66)	*
TI	3.47 (0.64)	3.41 (0.85)	3.32 (0.73)	–	2.60 (1.00)	2.25 (0.84)	2.44 (0.77)	**
P	4.58 (2.67)	3.75 (2.03)	3.65 (2.49)	***	7.65 (1.12)	2.88 (1.37)	3.3 (1.24)	***
JP	3.97 (0.55)	4.04 (0.65)	4.16 (0.76)	*	3.83 (0.54)	4.13 (0.73)	4.04 (0.59)	***
QH	3.98 (0.60)	3.75 (0.72)	4.01 (0.74)	***	3.71 (0.54)	3.95 (0.79)	3.98 (0.61)	**

Notes: Values are means (SD)

***P < 0.001, **P < 0.01, *P < 0.05

**Table 12:** Differences in all variables’ items among healthcare worker subgroups (part 2)

	Age			*P*	Job title		*P*	Sex		*P*	Seniority		*P*
	Young (*n* = 199)	Middle (*n* = 235)	Old (*n* = 127)		Early career (*n* = 251)	Mid/late career (*n* = 292)		Men (*n* = 200)	Women (*n* = 234)		< 5 years (*n* = 228)	> 5 years (*n* = 331)	
H	3.64 (0.80)	3.66 (0.77)	3.66 (0.86)	–	3.60 (0.78)	3.68 (0.82)	–	3.70 (0.81)	3.62 (0.78)	–	3.64 (0.80)	3.66 (0.80)	–
CS	2.40 (0.73)	3.42 (0.78)	3.42 (0.71)	–	3.37 (0.78)	3.45 (0.73)	–	3.41 (0.69)	3.43 (0.79)	–	3.39 (0.66)	3.43 (0.80)	–
HS	2.81 (0.88)	2.85 (0.95)	2.75 (0.75)	–	2.81 (0.88)	2.81 (0.88)	–	2.92 (0.85)	2.75 (0.91)	–	2.82 (0.87)	2.80 (0.89)	–
PSM	3.71 (0.80)	3.83 (0.76)	4.11 (0.83)	***	3.76 (0.78)	3.93 (0.83)	**	3.84 (0.77)	3.86 (0.82)	–	3.74 (0.81)	3.94 (0.78)	**
JS	3.62 (0.74)	3.67 (0.60)	3.80 (0.68)	–	3.64 (0.72)	3.74 (0.63)	–	3.66 (0.68)	3.71 (0.67)	–	3.67 (0.67)	3.69 (0.67)	–
TI	2.42 (0.87)	2.42 (0.89)	2.28 (0.83)	–	2.40 (0.88)	2.37 (0.84)	–	2.41 (0.84)	2.37 (0.87)	–	2.45 (0.84)	2.35 (0.89)	–
P	3.96 (1.95)	3.85 (2.11)	4.58 (2.88)	*	3.75 (1.96)	4.25 (2.46)	*	4.38 (2.52)	3.81 (2.06)	**	3.88 (2.06)	4.16 (2.39)	–
JP	3.89 (0.64)	4.06 (0.61)	4.20 (0.72)	***	3.93 (0.61)	4.12 (0.69)	**	3.97 (0.67)	4.07 (0.64)	–	3.90 (0.64)	4.12 (0.65)	***
QH	3.70 (0.64)	3.97 (0.70)	4.10 (0.89)	***	3.82 (0.64)	3.99 (0.73)	**	3.96 (0.68)	3.88 (0.70)	–	3.80 (0.62)	3.98 (0.73)	**

Notes: Values are means (SD)

***P < 0.001, **P < 0.01, *P < 0.05

### Change in healthcare worker status in Beijing public hospitals after reform

To measure the effect of reforms on the status of healthcare workers, we asked questionnaire respondents to rate variables before and after reform. We found that, after the reforms, 40.9% thought their health had improved (Table 13), 37.5% and 48.2% thought that challenge and hindrance stress had decreased, 17.7% thought their PSM had increased, 54.4% thought their job satisfaction had increased, 61.4% thought their turnover intention had diminished, 37.2% thought their presenteeism had decreased, 61.1% thought their job performance had increased, and 56.3% thought that quality of healthcare had improved.

**Table 13:** Changes in the status of healthcare workers in Beijing public hospitals

Variables	Percentages
H	(1) Greatly improved (5.6%); (2) Improved somewhat (35.3%); (3) No change (48.6%); (4) Deteriorated somewhat (8.2%); (5) Greatly deteriorated (2.1%)
CS	(1) Greatly reduced (8.4%); (2) Reduced somewhat (29.1%); (3) No change (48.4%); (4) Increased somewhat (8.8%); (5) Greatly increased (5.4%)
HS	(1) Greatly reduced (12.8%); (2) Reduced somewhat (35.4%); (3) No change (42.6%); (4) Increased somewhat (5.3%); (5) Greatly increased (3.9%)
PSM	(1) Greatly raised (3.8%); (2) Raised somewhat (13.9%); (3) No change (76.4%); (4) Reduced somewhat (4.1%); (5) Greatly reduced (1.8%)
JS	(1) Greatly raised (13.5%); (2) Raised somewhat (40.9%); (3) No change (36.7%); (4) Reduced somewhat (6.4%); (5) Greatly reduced (2.5%)
TI	(1) Greatly raised (4.6%); (2) Raised somewhat (13.1%); (3) No change (20.9%); (4) Reduced somewhat (42.8%); (5) Greatly reduced (18.6%)
P	(1) Greatly raised (9.7%); (2) Raised somewhat (12.9%); (3) No change (40.2%); (4) Reduced somewhat (31.5%); (5) Greatly reduced (5.7%)
JP	(1) Greatly raised (21.4%); (2) Raised somewhat (39.7%); (3) No change (24.8%); (4) Reduced somewhat (10.2%); (5) Greatly reduced (3.9%)
QH	(1) Greatly improved (13.5%); (2) Improved somewhat (42.8%); (3) No change (34.6%); (4) Reduced somewhat (6.7%); (5) Greatly reduced (2.7%)

*H* health, *CS* challenge stress, *HS* hindrance stress, *PSM* public service motivation, *JS* job satisfaction, *TI* turnover intention, *P* presenteeism, *JP* job performance, *QH* quality of healthcare

## Discussion

To determine the status of healthcare workers after comprehensive reform of urban public hospitals, we surveyed 572 healthcare workers from public primary, secondary, and tertiary level hospitals in Beijing.

Efforts to ensure a sustainable supply of healthcare workers in Beijing public hospitals faced challenges. On the one hand, the health of healthcare workers in Beijing public hospitals was worse than expected. The present respondents reported health problems such as pain, anxiety, depression, irritability, lack of energy, and difficulty walking, especially those working in public tertiary hospitals. Unfortunately, their health was not significantly improved by the comprehensive reform of urban public hospitals. Almost half (48.6%) reported no change in health after reform, and 10.3% reported worse health.

On the other hand, there is a substantial gap between the demand and supply of healthcare workers, and the poor health status of the respondents are likely attributable to the insufficient supply of such workers in Beijing public hospitals. Although the number of healthcare workers in these hospitals increased from 140 304 in 2011 to 204 048 in 2017, the average annual growth rate and annual number of newly licensed physicians have decreased since reform. At the end of 2017, the numbers of licensed (assistant) physicians (60 395) and registered nurses (85 785) were small and lower than the number of hospital beds (117 867), while the numbers of outpatients and inpatients reached 160.8 million and 3.69 million, respectively. The inadequate supply of healthcare workers is likely due to the poor working environment and perceived imbalance between effort and reward. Frequent incidents of violence toward healthcare workers, long working hours, heavy workloads, and unsatisfactory incomes make work in medicine and health care challenging [16]. Large-scale surveys of healthcare workers reported that only 24% of participants would choose the profession if they had a second chance and that more than 60% would not encourage their child to become a physician [30]. Chinese universities have produced 4 727 977 medical graduates over the past 10 years, but the total number of registered physicians in clinical practice increased by only 752 233 (15.91%) [31]. Therefore, the State Council of China developed a long-term plan to train and supply additional qualified medical professionals, in 2017 [16], and the shortage of healthcare workers in Beijing public hospitals is expected to end in the future.

Healthcare workers in Beijing public hospitals had strong PSM but faced extreme challenge stress. As public servants, healthcare workers in Beijing public hospitals attach great importance to public values and devote themselves to safeguarding public interests, which was consistent with our predictions and with previous findings [32]. In addition, among healthcare workers in public tertiary hospitals, those who are older, in mid/late career, and those with more than 5 years of seniority had relatively high PSM, which suggests that employment in a public organization has a positive effect on PSM, thus partially confirming the effect of organizational factors on PSM [33].

PSM is believed to help workers actively cope with job stress [34]. However, the present respondents reported extreme challenge stress due to their workload, medical violence, and job responsibilities. A previous study reported that 78% of physicians worked longer than 8 h a day and that 7% worked longer than 12 h a day [16]. Moreover, patient violence toward healthcare workers had nearly doubled: from 9831 in 2006 to 17 243 in 2010 [9]. One study found that 50% of medical professionals had experienced workplace violence during the previous 12 months and that 8% of hospitals had reported six or more incidents of physical violence every year [35, 36]. In addition, the job responsibilities of healthcare workers in public hospitals are complex and typically include medical, research, teaching, and administrative duties. Moreover, healthcare reform might have increased job stress among healthcare workers [37], because of changes in work content and processes and additional taskwork. High challenge stress may diminish its positive effects and even produce negative results.

Healthcare workers in Beijing public hospitals had relatively high job satisfaction and low turnover intention. As was reported in previous studies [37], we hypothesized that healthcare workers in hospitals undergoing reform would have high job satisfaction, which was confirmed in the present study. Interestingly, job satisfaction was higher among healthcare workers in public tertiary hospitals than among those in public secondary and public primary hospitals. This suggests that healthcare reform of the personnel and management systems has yielded benefits. However, healthcare workers remained dissatisfied with some aspects of their employment, e.g., compensation. The average annual income of physicians in 2015 was 77 000 RMB (about 12 360 USD), in contrast to an average annual income of 294 000 USD for physicians in the United States [16]. This salary discrepancy obviously does not reflect differences in relative workloads or work value. Moreover, increases in medical negligence and negative news coverage have diminished the professional image and social identity of physicians.

We found that healthcare workers preferred to stay in their present job rather than look for a new job, especially those in public secondary hospitals. This result may reveal the special reason for the characteristics of healthcare workers’ career choice in China. Public hospitals in China are much more developed than private hospitals and receive substantial financial and policy support from the government.

Therefore, work outcomes were good for healthcare workers in Beijing public hospitals, although they did report challenges. Because healthcare workers have to satisfy growing demand for services, job performance and quality of healthcare were high, which indicates that reform has greatly improved performance evaluations and quality management. However, work outcomes significantly differed among healthcare workers: namely, job performance and quality of healthcare were positively associated with hospital level, age, job title, and seniority.

Healthcare workers had moderate presenteeism (indicating loss of work ability), which threatens job performance and healthcare quality. Health and job stress are considered the main antecedents of presenteeism. Unfortunately, healthcare workers in Beijing public hospitals had low health status and high job stress. Therefore, the present strong job performance and good quality of healthcare might not be sustainable unless new reforms are introduced.

Lastly, the effects of reform on healthcare workers were not homogeneous. The status of healthcare workers in Beijing public hospitals generally improved after reform, although some negative factors persist. Positive evaluation of variables varied from 17.7 to 61.4%, and improvements in job satisfaction, turnover intention, job performance, and quality of healthcare were significant. Improvements in health, job stress, and presenteeism were only moderate and need to be highlighted in future reforms. Improvement in PSM was also modest, perhaps because the factors that underlie motivation are deep and thus change more slowly. Our analysis suggests that, although measures implemented by public hospitals to improve the status of healthcare workers have had positive effects, deficiencies remain. Indeed, some reform measures have not yet been properly implemented or have not yet shown benefits. Moreover, the content and difficulty of reforms substantially differ at primary, secondary, and tertiary hospitals, which could limit the effectiveness of some reforms.

The findings of this study should encourage policy makers to emphasize humanistic care of healthcare workers in future reform. First, salary and performance evaluation systems must be adjusted to reflect the true value of the labor of healthcare workers. Second, job stress should be reduced for healthcare workers by improving diagnosis, treatment, and referral systems and by encouraging patients with common and chronic diseases to visit public primary hospitals and community hospitals. Third, the health and well-being of healthcare workers must be maintained by encouraging them to seek annual physical examinations and by providing them with adequate medical insurance. Fourth, enacting laws against violence toward healthcare workers would increase job satisfaction.

### Limitations

To our knowledge, this is the first study of the status of healthcare workers after the comprehensive reform of urban public hospitals in Beijing. However, several limitations warrant attention. First, we asked participants to evaluate changes in selected critical variables after reform but did not examine changes before or after reform. Therefore, longitudinal effects on healthcare workers should be examined in future studies. Second, data on health, job stress, and other variables were self-reported. Therefore, respondents might provide socially desirable responses on self-completed questionnaires. Future studies should collect objective data as part of the study design. Third, although no new policy has been introduced since the reforms, the status of healthcare workers could have been affected by factors other than healthcare reform. Therefore, future studies should empirically investigate the effects of other important factors.

## Conclusion

The present analysis of statistical, questionnaire, and interview data shows that the number of healthcare workers in Beijing public hospitals has gradually increased. Healthcare workers in Beijing public hospitals scored higher in challenge stress, PSM, job satisfaction, presenteeism, job performance, and quality of healthcare but lower in health, hindrance stress, and turnover intention. In addition, the status of healthcare workers changed after comprehensive reform of urban public hospitals in Beijing. To provide superior healthcare services and promote reform throughout the country, policymakers and public-hospital administrators must pay greater attention to the welfare of healthcare workers by improving the salary and performance evaluation systems, reducing job stress, emphasizing the health of healthcare workers, and improving their work environment.

## Endnotes

1. The data of Table 2 on the number of healthcare workers, hospital beds, and outpatient and inpatients can be found at the Beijing Municipal Health Commission Information Center, at http://www.phic.org.cn/tjsj/wstjjb/

## Data Availability

The data sets used and/or analyzed during the current study are available from the corresponding author on reasonable request.

## Abbreviations

PSM: Public service motivation
SD: Standard deviation
